# Post-traumatic stress disorder, depression and generalised anxiety disorder in adolescents after a natural disaster: a study of comorbidity

**DOI:** 10.1186/1745-0179-2-17

**Published:** 2006-07-26

**Authors:** Nilamadhab Kar, Binaya Kumar Bastia

**Affiliations:** 1 Consultant Psychiatrist, Corner House Resource Centre, 300, Dunstall Road, Wolverhampton, WV6 0NZ, UK; 2 Binaya Kumar Bastia. Associate Professor, Forensic Medicine, SDM College of Medical Sciences, Sattur, Dharwad, Karnataka, India

## Abstract

**Background:**

Information on mental health sequel in adolescents following natural disasters from developing countries is scant.

**Method:**

Around one year after a super-cyclone, proportion of adolescents exhibiting post-traumatic psychiatric symptoms, prevalence of post-traumatic stress disorder (PTSD), major depression and generalized anxiety disorder, comorbidity and impairment of performance in school were studied in Orissa, India. Mini International Neuropsychiatric Interview for children and adolescents was used for evaluation and diagnosis. The criteria for diagnoses were based on Diagnostic and Statistical Manual of Mental Disorders – IV.

**Results:**

Post-disaster psychiatric presentation in adolescents was a conglomeration of PTSD, depression and anxiety symptoms. The prevalences of PTSD, major depressive disorder and generalised anxiety disorder were 26.9%, 17.6% and 12.0% respectively. Proportion of adolescents with any diagnosis was 37.9%. Comorbidity was found in 39.0% of adolescents with a psychiatric diagnosis. Adolescents from middle socioeconomic status were more affected. There were gender differences in the presentation of the symptoms rather than on the prevalence of diagnoses. Prolonged periods of helplessness and lack of adequate post-disaster psychological support were perceived as probable influencing factors, as well as the severity of the disaster.

**Conclusion:**

The findings of the study highlight the continuing need for identification and intervention for post-disaster psychiatric morbidities in adolescent victims in developing countries.

## Background

Reported rates of prevalence of post-disaster psychiatric disorders in children and adolescents vary across studies. Following Hurricane Hugo in Berkeley County, South Carolina, more than 5% school-aged children had post-traumatic stress syndrome at three months [1]; and after one year the rates of post-traumatic stress disorder (PTSD) ranged from 1.5% in black males to 3.8–6.2% in the remaining groups [2]. Approximately 3% of males and 9% of females met the criteria for PTSD six months after Hurricane Andrew in Dade County, Florida; rates were highest among blacks (8.3%) and Hispanics (6.1%) [3]; and 21 months after it 70% of the children had moderate-severe post-traumatic stress symptomatology [4]. Following Northridge Earthquake in Los Angeles, 28.5% of children had mild to moderate PTSD at one year [5]. Two years after Buffalo Creek dam collapse in 1972 approximately 37% of the children were given a "probable" diagnosis of PTSD [6]. Three and a half years after the Marmara earthquake in Turkey 22.2% adolescents had probable PTSD and 30.8% had probable depression diagnoses [7].

It has been observed that psychiatric morbidity among children and adult survivors of massive disasters in the Third World far exceeds those found following disasters in United States communities [8]. Studies from south Asian countries are almost non-existent even though the frequency and the extent of natural disasters in this part of the world are considerable. As trauma during childhood and adolescents can etch an indelible signature in the individual's development and may lead to future disorders [9], it underscores the need for such studies.

A super-cyclone with a wind speed of 260 to 300 kilometer per hour hit Orissa, in the East Coast of India, and continued for 72 hours from October 29, 1999. Tidal waves from sea at a height of 7 meters came 15 kilometers inland at various places and swept away almost everything. It affected over 15 million people; around 10,000 persons were killed; and there was massive loss to properties and livelihood. Thousands of villages were marooned for over two weeks before they could gain access to relief services [10-12].

Reports suggest that post-disaster psychiatric morbidity in adolescents continues for years [7]. The prevalence differ depending upon various factors e.g. severity of exposure, duration from the event and the methodology used [13,14], besides the differential response to trauma depending upon developmental level [15]. However studies have noted that parents, teachers and even mental health professionals significantly underestimate both the intensity and the duration of the stress reactions in children [16].

There is suggestion that children and adolescents present different type of psychopathology following disasters which are characterized by symptoms of post-traumatic stress disorder, depression, anxiety, aggressive and regressive behaviours. There is expanding literature regarding comorbidity of anxiety and depression in children and adolescents [8,17]. Depressive disorders have been found to be associated with the co-occurrence of anxiety and conduct disorders. Symptoms of both depression and anxiety have been noted to be more severe when these condition co-occur [18].

The present study had following specific objectives: to find (i) proportion of adolescents exhibiting post-traumatic psychiatric symptoms, (ii) prevalence of PTSD, major depressive disorder (MDD), and generalized anxiety disorders (GAD), and (iii) prevalence of comorbidity in a group of adolescent students in rural areas, 14 months after the super-cyclone of Orissa. Associated sociodemographic factors and effect of morbidity on performance in school were also studied.

## Methods

All the students studying in standard nine and ten of two high schools in the most severely affected Jagatsinghpur district were taken as subjects of the study. Mini International Neuropsychiatric Interview for children and adolescents (MINI-KID) was used for evaluation of symptoms and diagnosis of MDD, PTSD and GAD [19]. English is taught in schools to all high school students; however we provided a translated version of the scale in the vernacular Oriya along with the English version. This was considered appropriate considering regional variance in expression of symptoms and understanding the essence of the concept. MINI-KID allows for the explanation of words and concepts in the questions if the child or adolescent does not understand a particular symptom. The process of preparation of Oriya version involved translation to Oriya and retranslation to English. It was piloted before the study.

The diagnoses were based on the Diagnostic and Statistical Manual of Mental Disorders – Fourth edition (DSM-IV) criteria [20]. Family structure (nuclear or joint), socioeconomic status (SES) following local guidelines, educational background of the parents and main earning source for the family were noted. Information on failures in examination and report of difficulties in studies were collected.

Data collection was done 14 months after the super-cyclone. Adolescents, their teachers and parents were briefed about the study. Informed consent was collected from parents or the responsible guardian. The adolescents were evaluated in their school. A parent or teacher was present during the clinical evaluation. The questionnaire was administered individually by one of the authors (BB). The study protocol was approved by the school managements and the ethics committee of Quality of Life Research and Development Foundation. Chi-square test was used to test association between categorical variables. Significance value was set at 0.05.

## Results

The sample consisted of 108 adolescents, constituting 60 females and 48 males with mean age of 14.3 ± 0.65 and 14.25 ± 0.83 years respectively. There was no difference between the genders regarding the family structure, first or subsequent born, or parental educational level. Main family income source for most male students (70.8%) was cultivation, whereas relatively more females (56.7%) were from families with other main sources of income (χ^2^: 8.2, df: 1, p: 0.004). There were 11.1% adolescents in lower SES, 80.6% in middle and 8.3% in upper SES. Most of the males (95.8%) were from middle SES compared to 68.3% females (χ^2^: 13.5, df: 1, p: 0.001).

All adolescents reported significant damage to their houses (making them uninhabitable), starvation, and lack of treatment for physical ailments in the initial few days after the cyclone. They witnessed the devastated scene with dead and mutilated bodies of human beings and cattle, damaged houses, uprooted trees after the cyclone. The victims had to depend on outside relief for months; some of them had to live in the shelters for weeks to months. None of the adolescents had exposure to any other psychiatric evaluation or formal intervention before the study.

### Post-traumatic psychiatric manifestations

A considerable number of adolescents reported various post-traumatic psychiatric symptoms. Symptoms whose prevalence was significantly different in adolescents with any psychiatric disorder (cases) from those with no diagnosis (non-cases) are presented in table 1. The prevalences of PTSD, MDD and GAD syndromes are given in table 2.

**Table 1:** Prevalence of post-traumatic psychiatric symptoms which significantly differentiated cases from non-cases

Symptoms		Diagnostic status		
	Total	Non cases	Cases	Statistics

	N = 108	n = 67	n = 41	χ^2^, df, p

Trouble remembering some important aspects of disaster	66.7	56.7	82.9	7.86, 1, 0.005
Felt that life will be shortened, die sooner than others	56.5	46.3	73.2	7.49, 1, 0.006
Avoidance	53.7	44.8	68.3	5.66, 1, 0.017
Trouble paying attention	52.8	44.8	65.9	4.53, 1, 0.033
Nervous or 'jumpy'	45.4	37.3	58.5	4.62, 1, 0.032
Dreaming, vivid memory about disaster	42.6	25.4	70.7	21.4, 1, 0.000
Anhedonia, lack of interest	41.7	31.3	58.5	7.74, 1, 0.005
Guilt feeling, feeling bad about self	37.0	28.4	41.2	5.7, 1, 0.017
Feel tense	36.1	28.4	48.8	4.59, 1, 0.03
Feel grouchy or annoyed	35.2	25.4	51.2	7.45, 1, 0.006
No strong feeling about things	35.2	26.9	48.8	5.36, 1, 0.02
Trouble making up mind	32.4	22.4	48.8	8.09, 1, 0.004
Less interested in hobbies or friends	30.6	23.9	41.5	3.71, 1, 0.054*
Tiredness most of the time	30.6	17.9	51.2	13.29, 1, 0.000
Trouble sleeping	29.6	20.9	43.9	6.45, 1, 0.011
Worrying most days	23.1	7.5	48.8	24.4, 1, 0.000
Death wish, self harm or suicidal ideas	21.3	11.9	36.6	9.21, 1, 0.002
Trouble sleeping in past month	19.4	10.4	34.1	9.12, 1, 0.003
Trauma related symptoms upset or cause problem	15.7	6.0	31.7	12.7, 1, 0.000

Figures indicate percentages of adolescents presenting the symptom. *approaching significance

**Table 2:** Prevalence of diagnoses and comorbidities in genders

Diagnoses		Female (n = 60)		Male (n = 48)		Total (N = 108)	
		n	%	n	%	N	%

PTSD		13	21.7	16	33.3	29	26.9
MDD		13	21.7	6	12.5	19	17.6
GAD		6	10.0	7	14.6	13	12.0
Any diagnosis		23	38.3	18	37.5	41	37.9
Comorbidity	no (one diagnosis)	16	26.7	9	18.8	25	23.1
	Two diagnoses	5	8.3	7	14.6	12	11.1
	Three diagnoses	2	3.3	2	4.2	4	3.7

No statistical significance

### Adolescents with psychiatric disorders

The number of adolescents with any of the three diagnoses studied was 41 (37.9%). Comparison of sociodemographic variables between cases with non-cases revealed that there was no difference between them in their family background, income source of family, birth order and education of father and mother. Most cases (92.7%) belonged to middle SES (χ^2^: 6.27, df: 2, p: 0.043). While the change of economic status was minimal for those in low SES; and persons in upper SES had additional resources for support; it was the middle SES families who were the hardest hit.

Psychopathologies which significantly differentiated the cases from non-cases were a mixture of depression, anxiety and post-traumatic stress symptoms. Cases had following PTSD symptoms significantly more: dreams, vivid memory, avoidance, trouble remembering few aspects of disaster, did not have strong feeling about things, felt that life would be shortened and they would die soon, sleep problems, trouble paying attention, nervousness, and remaining upset about these symptoms. Depressive symptoms which were associated significantly with the psychiatric caseness were decreased interest and anhedonia, tiredness, guilt feeling, impaired concentration and death wish or suicidal ideas. Similarly, adolescents with any psychiatric diagnosis had following GAD symptoms significantly more: worrying most days, feeling tense, grouchy or annoyed and trouble sleeping.

Educational impairment was comparable amongst the diagnoses studied and between cases and non-cases. According to teachers, there was a perceptible decrease in motivation and perceived purposelessness in studying amongst students; and attending school had become an activity to spend time rather than being there to study.

### Comorbidity

Sixteen adolescents (39.0%) out of 41 who had any psychiatric diagnosis had comorbidity. Prevalence of comorbidity in adolescents with PTSD was 48.3%; it was 63.2% with MDD, and 76.9% with GAD. Six adolescents had MDD and PTSD, 4 had PTSD and GAD, 2 had GAD and MDD and 4 had all three diagnoses.

The comorbidity was not only due to the symptomatic overlap amongst these diagnoses, but considerable prevalence of various core symptoms of each of these diagnoses were also observed in others. For example, following depressive symptoms were significantly present in adolescents who had PTSD compared to those who did not have PTSD: anhedonia (62.1% vs. 34.2%; χ^2^: 6.79, df: 1, p: 0.009; tiredness (51.7% vs. 22.8%; χ^2^: 8.37, df: 1, p: 0.004), death wish and suicidal ideas (34.5% vs. 16.5%; χ^2^: 4.11, df: 1, p: 0.04). Anxiety symptoms that were significantly present in adolescents with PTSD were worry (93.1% vs. 75.9%; χ^2^: 3.98, df: 1, p: 0.046) and difficult to stop worrying (93.1% vs. 73.4%; χ^2^: 4.9, df: 1, p: 0.027). Adolescents with MDD had the following PTSD symptoms significantly more compared to those without MDD: dreams (68.4% vs. 37.1%; χ^2^: 6.29, df: 1, p: 0.012), avoidance (78.9%, vs. 48.3%; χ^2^: 5.91, df: 1, p: 0.015), not having strong feeling about things (63.2% vs. 29.2%; χ^2^: 7.9, df: 1, p: 0.005); and following anxiety symptoms: worrying most days (52.6% vs. 16.9%; χ^2^: 11.26, df: 1, p: 0.001), feeling not able to sit still (57.9% vs. 30.3%; χ^2^: 5.21, df: 1, p: 0.02), feeling tense (57.9% vs. 31.5%; χ^2^: 4.74, df: 1, p: 0.029), grouchy and annoyed (57.9% vs. 30.3%; χ^2^: 5.2, df: 1, p: 0.02). It appeared that sleep problem was common to all.

Adolescents with comorbidity were significantly differentiated from the ones with only one diagnosis by the following symptoms: decrease concentration (68.8% vs. 36.0%, χ^2^: 4.19, df:1, p: 0.041), not having strong feeling about things (81.3% vs. 28.0%, χ^2^: 11.1, df: 1, p: 0.001), worry (100.0% vs. 76.0%, χ^2^: 4.49, df: 1, p: 0.034), worrying most days (68.8% vs. 36.0%, χ^2^: 4.19, df: 1, p: 0.041) and feeling tense (81.3% vs. 28.0, χ^2^: 11.1, df: 1, p: 0.001). Depressed mood was present in 98.8% of adolescents with comorbidity compared to 68.0% of those without (χ^2^: 3.78, df: 1, p: 0.05).

### Gender differences in psychiatric morbidity

There were no significant differences in the prevalence of diagnoses in male and female adolescents (table 2). There were a few differences in their presentation of symptoms. Considering PTSD symptoms significant difference was found in dreams about the disaster and having strong memory which was more in males than in females (54.2% vs. 33.3% respectively, χ^2^: 4.73, df: 1, p: 0.03); similarly more males had avoidance and tried not to think about it (66.7% vs. 43.3%, χ^2^: 5.84, df:1, p: 0.016) and felt cut off from other people (37.5% vs. 11.7%, χ^2^: 10.0, df: 1, p: 0.002). More females (58.3%) than males (39.6%) reported startle reactions which approached statistical significance (χ^2^: 3.75, df: 1, p: 0.053).

Symptoms of GAD were mostly uniformly distributed except that more males compared to females felt it hard to stop worrying (87.5% vs. 71.7%, χ^2^: 3.98, df: 1, p: 0.046) and had trouble falling asleep and waking up in the middle of night (41.7% vs. 20.0%, χ^2^: 6.0, df: 1, p: 0.014).

Studying the gender differences in depressive symptoms, it was observed that compared to females significantly more numbers of males reported decreased interest or anhedonia (28.3% vs. 58.3%, χ^2^: 9.87, df: 1, p: 0.002), change in appetite or weight (41.7% vs. 62.5%, χ^2^: 4.63, df: 1, p: 0.031), and lack of concentration (18.3% vs. 50.0%, χ^2^:12.2, df: 1, p: 0.000). Significantly more number of females reported guilty feeling or feeling bad about self (51.7% vs. 18.8%, χ^2^: 12.4, df: 1, p: 0.000) compared to males.

There were considerable differences between the genders on school performance. More males reported difficulty in studies (47.9% vs. 28.3%, χ^2^: 4.4, df: 1, p: 0.036) and failure in examinations (29.2% vs. 13.3%, χ^2^: 4.1, df: 1, p: 0.04) than females.

## Discussion

The study demonstrated that adolescents exposed to super-cyclone exhibited a wide range of post-traumatic symptoms. A considerable proportion of them fulfilled the diagnosis parallel to DSM-IV criteria [20] after 14 months of the natural disaster. Comorbidity was common.

PTSD was the most common diagnosis; additionally a considerable proportion had MDD and GAD. Prevalence of PTSD was comparable to that reported after Northridge Earthquake [5] but higher to that following Hurricane Hugo [2], both at one year post-disaster period. Variations in rates for specific psychopathologies were also noted. One year after Hurricane Hugo, re-experiencing was reported by 20% of adolescents [2] compared to 42.6% in our study; and avoidance was reported by 9% compared to 53.7% respectively. Death wish, self-harm or suicidal ideas was present in 21.3% of adolescents in our study which is comparable to that reported else where [8]. However comparison of prevalence rates of psychiatric morbidity following different disasters might be difficult considering many factors. Multiple variables like nature and intensity of the trauma, personal loss, individual vulnerabilities, post disaster adversities and psychosocial support, besides the methods of evaluation and period passed since the disaster etc. are known to influence the prevalence.

It becomes imperative to discuss the vulnerability of the subjects studied in view of the level of morbidity observed. The super-cyclone affected a large population. Communication got cut off and it took long to re-establish it following the disaster. Any kind of initial help and support reached victims only days after and in some places even weeks as vast areas were affected. It prolonged the period of helplessness. Most of the victims belonged to lower and middle socioeconomic strata, with cultivation or manual labour as the main source of earning. Following disasters these resources got completely damaged; and people could only hope for external support. It was perceived that support did not match up for the need, nor did it continue for periods they were needed. So the secondary traumas continued, without work and rehabilitation. Post-disaster adversities contributing to the psychological problems of the victims and delaying their recovery have been suggested [21]. Besides exposure to trauma and resource loss, continued stress is probably responsible for the higher morbidity in our sample.

It has been reported that post-disaster psychosocial support influences the psychiatric morbidity [22,1]. Subjects of this study received no formal psychological support. As meeting basic needs was the obvious priority following disaster and the external support struggled to meet these demands, the mental health needs of the victims could never come to focus. There was no way obviously for the existing mental health care system with services only at tertiary level or in private practice, to meet the demands of post-disaster mental health problems. Psychological support through disaster workers was minimal if any, and was restricted by training, preparedness and resource issues in this developing state. It resulted in situations where there was no evaluation or management of psychiatric sequel following the disaster in most areas, which may be one of the reasons for high prevalence of psychiatric morbidity.

It may be highlighted that, in spite of all the adversities caused by the disaster and post-disaster situation, majority of adolescents were not having syndromal psychiatric disorders. Protective factors behind this need focused study. However, there is a possibility of many mechanisms of informal psychological support from close-knit social network in Indian villages.

### Comorbidity

The study results suggested that comorbidity was common in post-disaster psychiatric diagnoses. A considerable proportion (39.0%) of adolescent victims with syndromal diagnoses had comorbidity; this was most likely for the ones with GAD, followed by MDD. PTSD, the most common diagnosis in the post-disaster scenario, had comorbidity in around half. Depression was the common comorbid diagnosis of PTSD, being present in around one third (34.5%). There has been report of high correlation between post-traumatic stress symptomatology and depression following natural disasters with a range of 13 to 75% [8]. GAD was the comorbid diagnosis in 27.6% adolescents with PTSD. There was overlap of all these three diagnoses in 13.8%. The results underscore that in post-disaster psychiatric surveys it is important to look for comorbidities; as they may give a holistic picture of the reaction to catastrophic stresses like natural disasters and help in management plan of the victims.

### Gender differences

In our study females had more depression diagnosis and less PTSD compared to males; however these differences were not statistically significant. Many previous studies have failed to demonstrate gender differences [23,24]; while some have reported females are more likely than males to report symptoms of anxiety [25], and post-traumatic stress [8]. Significantly more females in our study reported guilt; and more males reported hard to stop worrying, anhedonia, concentration problems. Similar symptoms were reported to be significantly associated with males following Hurricane Hugo [1]. Reported gender differences in PTSD symptoms in children exposed to trauma have been variable across studies. It has been suggested that gender difference, if existent, may depend upon various factors, and may get obliterated by the higher exposure to trauma.

Problems with performance in school (difficulties in studies and failure in examinations) were significantly associated with males. It was not associated with caseness or with any particular diagnosis, which is in contrast to the finding of it being more than three times likely in children with PTSD related to Hurricane Hugo [1]. As information on the pre-trauma performance was not ascertained it is difficult to comment on the degree of decrease in performance in school following disaster in our study.

### Limitation

Absence of standardised diagnostic instrument in the vernacular Oriya is a limitation; however English is studied and commonly used by the study population, and translated version of the questionnaire was helpful regarding clarification of some symptoms. We did not have information on pre-cyclone psychiatric morbidity, which is known to influence the post-disaster psychiatric morbidity [5]. Influence of various other risk factors like death of family members, serious physical trauma and supportive factors were not noted. Comparative study with an unexposed control population could have suggested the risk attributable to cyclone and secondary traumas associated with it. The study focused only on three disorders. Presence of other disorders and comorbidities is a likely possibility in the studied population.

## Conclusion

A considerable proportion of adolescents suffered from stress related symptoms and had syndromal psychiatric diagnoses, around one year after the super-cyclone. Overlap of symptoms and comorbidity of diagnoses were high, suggesting that post-disaster presentation is often a conglomeration of PTSD, depression and anxiety symptoms. As victims continue to suffer from trauma related psychiatric disorders, long after the disaster, the need for screening and intervention continues to be there especially so when the mental health care has not been in place from the beginning along with other disaster related support.

## Competing interests

The author(s) declare that they have no competing interests.

## Authors' contributions

NK conceptualised, wrote protocol, analysed data, wrote the article, BB did clinical evaluation and collected data; both authors read and approved final manuscript.

## References

[B1] Shannon MP Lonigan CJ Finch AJ Jr Taylor CM Children exposed to disaster: I. Epidemiology of post-traumatic symptoms and symptom profiles J Am Acad Child Adolesc Psychiatry 1994 33 1 80 93 8138525 10.1097/00004583-199401000-00012

[B2] Garrison CZ Weinrich MW Hardin SB Weinrich S Wang L Post-traumatic stress disorder in adolescents after a hurricane Am J Epidemiol 1993 1; 138 7 522 530 10.1093/oxfordjournals.aje.a116886 8213756

[B3] Garrison CZ Bryant ES Addy CL Spurrier PG Freedy JR Kilpatrick DG Post-traumatic stress disorder in adolescents after Hurricane Andrew J Am Acad Child Adolesc Psychiatry 1995 34 9 1193 1201 10.1097/00004583-199509000-00017 7559314

[B4] Shaw JA Applegate B Schorr C Twenty-one-month follow-up study of school-age children exposed to Hurricane Andrew J Am Acad Child Adolesc Psychiatry 1996 35 3 359 364 10.1097/00004583-199603000-00018 8714325

[B5] Asarnow J Glynn S Pynoos RS Nahum J Guthrie D Cantwell DP Franklin B When the earth stops shaking: earthquake sequelae among children diagnosed for pre-earthquake psychopathology J Am Acad Child Adolesc Psychiatry 1999 38 8 1016 1023 10.1097/00004583-199908000-00018 10434494

[B6] Green BL Korol M Grace MC Vary MG Leonard AC Gleser GC Smitson-Cohen S Children and disaster: age, gender, and parental effects on PTSD symptoms J Am Acad Child Adolesc Psychiatry 1991 30 6 945 951 1757444 10.1097/00004583-199111000-00012

[B7] Karakaya I Agaoglu B Coskun A Sismanlar SG Yildiz Oc O The symptoms of PTSD, depression and anxiety in adolescent students three and a half years after the Marmara earthquake Turk Psikiyatri Derg 2004 15 4 257 263 15622505

[B8] Goenjian AK Pynoos RS Steinberg AM Najarian LM Asarnow JR Karayan I Ghurabi M Fairbanks LA Psychiatric comorbidity in children after the 1988 earthquake in Armenia J Am Acad Child Adolesc Psychiatry 1995 34 9 1174 1184 10.1097/00004583-199509000-00015 7559312

[B9] Schwarz E Perry BD The post-traumatic response in children and adolescents Psychiatric Clinics of North America 1994 17 311 326 7937362

[B10] Srinivasa Murthy R Kar N Sekar K Swain S Mishra V Daniel U Evaluation report on psychosocial care of survivors of supercyclone in Orissa 2003 SnehaAviyan, ActionAid Bhuabaneswar, NIMHANS, Bangalore

[B11] Juvva S Rajendran P Disaster mental health Current perspective Indian Journal of Social Work 2000 61 4 527 541

[B12] Kar N Jagadisha Sharma PSVN Murali N Mehrotra S Mental health consequences of the trauma of supercyclone 1999 in Orissa Indian Journal of Psychiatry 2004 46 228 237 PMC2951648 21224904

[B13] Murali N Kar N Jagadisha Recognition and clinical assessment of childhood PTSD Indian Journal of Psychiatry 2002 44 1 82 83 PMC2953662 21206889

[B14] Kar N Jagadisha Murali N Post-traumatic stress disorder in children following disaster Kerala Journal of Psychiatry 2001 16 2 7 14

[B15] Deering CG A cognitive developmental approach to understanding how children cope with disasters J Child Adolesc Psychiatr Nurs 2000 13 1 7 16 11022467 10.1111/j.1744-6171.2000.tb00070.x

[B16] American Academy of Child and Adolescent Psychiatry Practice parameters for the assessment and treatment of post-traumatic stress disorder in children and adolescents. Judith A Cohen, principal author J Am Acad Child Adolesc Psychiatry 1998 37 Suppl 4S 10.1097/00004583-199810001-00002 9785726

[B17] Angold A Costello EJ Depressive comorbidity in children and adolescents: empirical, theoretical and methodological issues Am J Psychiatry 1993 150 1779 1791 8238631 10.1176/ajp.150.12.1779

[B18] Strauss CC Last CG Hersen M Kazdin AE Association between anxiety and depression in children and adolescents with anxiety disorders Abnorm Child Psychol 1988 16 57 68 10.1007/BF00910500 3361030

[B19] Sheehan D Shytle D Milo K Lecrubier Y Herguetta T Mini International Neuropsychiatric Interview for children and adolescents http://www.medical-outcomes.com 1.1, January 1, 2000

[B20] American Psychiatric Association DSM-IV, Diagnostic and Statistical Manual of Mental disorders, Washington 1994 4

[B21] Goenjian A A mental health relief programme in Armenia after the 1988 earthquake. Implementation and clinical observations Br J Psychiatry 1993 163 230 239 8075916 10.1192/bjp.163.2.230

[B22] Lonigan CJ Shanon MP Taylor CM Finch AJ Sallee FR Children exposed to disasters: II. Risk factors for the development of post-traumatic symptomatology J Am Acad Child Adolesc Psychiatry 1994 33 1 94 105 8138526 10.1097/00004583-199401000-00013

[B23] Earls F Smith E Reich W Jung KG Investigating psychopathological consequences of a disaster in children: a pilot study incorporating a structured diagnostic interview J Am Acad Child Adolesc Psychiatry 1988 27 90 95 3343213 10.1097/00004583-198801000-00014

[B24] McFarlane AC Post-traumatic phenomenon in a longitudinal study of children following natural disaster J Am Acad Child Adolesc Psychiatry 1987 26 764 769 3667509 10.1097/00004583-198709000-00025

[B25] Burke JD Jr Moccia P Borus JF Burns BJ Emotional distress in fifth-grade children ten months after a natural disaster J Am Acad Child Psychiatry 1986 25 536 541 3745734 10.1016/s0002-7138(10)60014-3

